# Metabolic syndrome risk prediction in an Australian sample with first-episode psychosis using the psychosis metabolic risk calculator: A validation study

**DOI:** 10.1177/10398562241269171

**Published:** 2024-08-13

**Authors:** Scott B Teasdale, Oliver Ardill-Young, Rachel Morell, Philip B Ward, Golam M Khandaker, Rachel Upthegrove, Jackie Curtis, Benjamin I Perry

**Affiliations:** Discipline of Psychiatry and Mental Health, School of Clinical Medicine, 7800 UNSW Sydney, Kensington, NSW, Australia; Mindgardens Neuroscience Network, Randwick, NSW, Australia; Discipline of Psychiatry and Mental Health, School of Clinical Medicine, 7800 UNSW Sydney, Kensington, NSW, Australia; Schizophrenia Research Unit, South Western Sydney Local Health District and Ingham Institute of Applied Medical Research, Liverpool Hospital, Liverpool, NSW, Australia; Department of Psychiatry, 2152 University of Cambridge, Cambridge, UK; Centre for Academic Mental Health, Population Health Sciences, Bristol Medical School, 1980 University of Bristol, Bristol, UK; MRC Integrative Epidemiology Unit, Population Health Sciences, Bristol Medical School, 1980 University of Bristol, Bristol, UK; Institute for Mental Health and Centre for Human Brain Health, 1724 University of Birmingham, Birmingham, UK; Early Intervention Service, 1729 Birmingham Women’s and Children’s NHS Foundation Trust, Birmingham, UK; Discipline of Psychiatry and Mental Health, School of Clinical Medicine, 7800 UNSW Sydney, Kensington, NSW, Australia; Mindgardens Neuroscience Network, Randwick, NSW, Australia; Early Psychosis Programme, South Eastern Sydney Local Health District, Bondi Junction, NSW, Australia; Department of Psychiatry, 2152 University of Cambridge, Cambridge, UK and; Department of General Psychiatry, 4028 Cambridgeshire and Peterborough NHS Foundation Trust, Cambridge, UK

**Keywords:** mental disorders, psychosis, antipsychotic, metabolic syndrome, risk prediction, validation study

## Abstract

**Objective:**

To examine the accuracy and likely clinical usefulness of the Psychosis Metabolic Risk Calculator (PsyMetRiC) in predicting up-to six-year risk of incident metabolic syndrome in an Australian sample of young people with first-episode psychosis.

**Method:**

We conducted a retrospective study at a secondary care early psychosis treatment service among people aged 16-35 years, extracting relevant data at the time of antipsychotic commencement and between one-to-six-years later. We assessed algorithm accuracy primarily via discrimination (C-statistic), calibration (calibration plots) and clinical usefulness (decision curve analysis). Model updating and recalibration generated a site-specific (Australian) PsyMetRiC version.

**Results:**

We included 116 people with baseline and follow-up data: 73% male, mean age 20.1 years, mean follow-up 2.6 years, metabolic syndrome prevalence 13%. C-statistics for both partial- (C = 0.71, 95% CI 0.64–0.75) and full-models (C = 0.72, 95% CI 0.65–0.77) were acceptable; however, calibration plots demonstrated consistent under-prediction of risk. Recalibration and updating led to slightly improved C-statistics, greatly improved agreement between observed and predicted risk, and a narrow window of likely clinical usefulness improved significantly.

**Conclusion:**

An updated and recalibrated PsyMetRiC model, PsyMetRiC-Australia, shows promise. Validation in a large sample is required to confirm its accuracy and clinical usefulness for the Australian population.

The physical health disparities experienced by people living with severe mental illness such as schizophrenia and related psychoses are well known.^1^ Physical health diseases are the major drivers for the 13-15 year reduced life expectancy in people with severe mental illness compared to those without mental illness,^2,3^ with cardiometabolic risks frequently developing early in the course of a psychotic illness and its treatment.^4^ Cardiometabolic risk prediction algorithms are routinely used in the general population to encourage personalised treatment decisions with the aim of primary prevention of longer-term cardiometabolic outcomes.^5^ However, general population algorithms are unsuitable for a young person with psychosis as they likely underpredict risk in all young people and do not take into account risks associated with metabolically active psychotropic medications prescribed to manage psychotic disorders.^6^

The Psychosis Metabolic Risk Calculator (PsyMetRiC) was first developed and externally validated in young people with psychosis in the United Kingdom,^7^ with subsequent validations in Western Europe.^8^ In both studies, PsyMetRiC reliably predicted up-to-six-year risk of metabolic syndrome,^7,8^ an age-appropriate precursor to cardiovascular disease and all-cause mortality.^9^ PsyMetRiC could be a valuable tool for healthcare professionals working with young people with psychosis, to help them identify those at higher cardiometabolic risk, and more appropriately factor physical health into their treatment decisions. However, the transportability of PsyMetRiC to Australian mental health services cannot be assumed due to likely population differences in baseline health, demographic and socioeconomic factors, legislature, and health service delivery.

This study aimed to examine the performance of the PsyMetRiC algorithm in predicting risk of incident metabolic syndrome in an Australian sample of young people with first-episode psychosis.

## Methods

### Study design and setting

A retrospective chart audit was conducted among people who received treatment from a secondary care early psychosis programme in the South Eastern Sydney Local Health District (SESLHD) between the inception of electronic Medical Records (eMR) in 2014 and 2022. This study was approved by the South Eastern Sydney Local Health District Human Research Ethics Committee (2021/ETH01397) using data from routine clinical care with a waiver of participant consent for health service evaluation in line with the New South Wales Legislation: Health Records and Information Privacy Act 2002 No. 71 [Section 10 (d)]. This study adhered to the TRIPOD reporting guidelines for prognosis research studies, see Supplementary Data for a completed TRIPOD checklist.^10^

### Participants

Included participants: (i) were aged 16-35 years when they first received a diagnosis of first-episode psychosis, (ii) were being treated by the SESLHD Mental Health Service, (iii) had anthropometric and metabolic measures taken ±100 days from diagnosis of first-episode psychosis, (iv) did not meet criteria for metabolic syndrome at baseline according to International Diabetes Federation (IDF) consensus guidelines,^11^ (v) had follow-up anthropometric and metabolic measures between 1 and 6 years from baseline, (vi) had necessary demographic and clinical information available.

### Outcome measure

IDF-consensus defined metabolic syndrome^11^ was calculated from follow-up data including waist circumference or body mass index (BMI), blood pressure, triglycerides, high-density lipoprotein (HDL) and fasting plasma glucose. See Supplemental File Part A for details on the collection of anthropometry, biochemistry and blood pressure data.

### Exposures – PsyMetRiC full and partial models

PsyMetRiC consists of two forced-entry multivariable penalised logistic regression equations: the full-model and the partial-model. The full-model includes age, sex, ethnic background, current smoking status, psychotropic medication at baseline, BMI, HDL and triglycerides. The partial-model is the same minus the biochemical predictors (HDL and triglycerides). Briefly, predictors were selected based on clinical knowledge, prior research, and likely clinical usefulness/patient acceptability. The partial-model was developed to cover situations where biochemical results may not be available. The PsyMetRiC algorithm coefficients are presented in Table S1. Antipsychotic medications were categorised as more or less metabolically active (see Supplemental File Part A & Table S2). See the original PsyMetRiC study^7^ for further details.

## Statistical analysis

### Sample preparation and estimation of analytic precision

We assessed for the presence of predictor multi-collinearity by measuring the variance inflation factor (Supplementary File: Table S5). We applied criteria^12^ to estimate analytic precision given the fixed sample size (Supplementary File Part A). Multiple imputation using chained equations was considered for missing data (Supplemental File Part A). For numerical-based analyses, estimates were pooled using Rubin’s rules. For plot-based analyses, plots were generated in each imputed dataset and checked for similarity, with one randomly selected plot per analysis presented in the main manuscript and all remaining plots presented in the Supplementary File. Comparisons between the original PsyMetRiC development sample and the Australian sample for key sociodemographic, lifestyle and biochemical characteristics were performed using t-tests (for means) and the chi-square equality of proportions test (for proportions).

### Primary external validation analysis

The distribution of predicted outcome probabilities was inspected using histograms. Algorithm performance was primarily assessed with measures of discrimination (concordance (C-) statistic), and calibration (calibration plots) (explained in Supplementary File: Part A). We also recorded the Nagelkerke-Cox-Snell-Maddala-Magee r^2^ index, the calibration intercept (ideally close to 0), calibration slope (ideally close to 1), and the Brier score (ideally close to 0, with scores >0.25 indicating poor performance). Given that the ethnic make-up of the Australian population is different to that of the UK, we were unable to use the existing PsyMetRiC ethnicity predictor in the primary external validation analysis, and so predictive performance was assessed without ethnicity information.

### Generation of site-specific PsyMetRiC version

Given the challenges of external validation in international samples, primarily population differences in distribution of predictors, we expected a difference in calibration performance compared with the original PsyMetRiC study, which was developed in the UK. In addition, given that the ethnic make-up of the Australian population is different to that of the UK, we were unable to use the existing PsyMetRiC ethnicity predictor. Therefore, we performed a model updating and recalibration approach to generate a site-specific version of PsyMetRiC and included the variable ‘Born in Australia’ or ‘Born outside of Australia’ instead of ethnicity (Supplementary File: Methods). We present performance estimates accompanied by 95% CIs (derived from an alpha-value of 0.05 as commonly used in inferential statistics).

### Clinical usefulness

Decision curve analysis^13^ was used to assess likely clinical usefulness by estimating net benefit across a range of feasible thresholds (i.e. the risk score at which an intervention would be deemed necessary by patient/clinician) (explained in Supplementary File: Methods). We considered a risk threshold upper-bound of 0.30, which represents around a one-in-three chance of developing metabolic syndrome should nothing change, because it is unlikely that risk thresholds greater than that would be tolerated without intervention.

### Data visualisation

An online data visualisation website for PsyMetRiC was created to accompany the original study (https://psymetric.shinyapps.io/psymetric/). The website was updated with site-specific (Australian) PsyMetRiC versions obtained through updating and recalibration analysis.

## Results

### Study sample

A total of 116 young people met inclusion criteria for this study. Table 1 details baseline sociodemographic, clinical, and biochemical characteristics of this sample and the original UK sample. Participants had a mean age of 20.1 years (SD = 3.2), were mostly born in Australia (67%), male (73%), non-smoker (65%), and were prescribed an antipsychotic drug with higher metabolic activity (84%). The Australian sample differed from the original UK PsyMetRiC development sample on most sociodemographic, clinical and biochemical characteristics. Mean follow-up time was 2.6 years (95% CI 2.3 to 2.8; range 1 to 6 years). Fifteen people (13%) met criteria for metabolic syndrome at follow-up.

**Table 1.** Baseline demographic, clinical and physical health details of participants compared with original PsyMetRiC development sample (UK).

Domain	Measurement	Original PsyMetRiC development sample (UK) (*n* = 651)	Analytic sample (*n* = 116)	Between-sample differences^c^
Demographic				
Age (years)	Mean ± SD	24.5 ± 4.9	20.1 ± 3.2	t = 9.3, *p* < 0.001
Male	*n* (%)	440 (55)	85 (73)	χ = 13.1, *p* < 0.001
Country of birth	*n* (%)			
Born in Australia		-	78 (67)	-
Born outside of Australia			38 (33)	
Smoker	*n* (%)	315 (48)	41 (35)	χ = 6.7, *p* = 0.001
Medications	*n* (%)			
Antipsychotic with higher metabolic activity potential		455 (70)	97 (84)	χ = 9.6, *p* = 0.002
Mood stabiliser		-	16 (14)	-
Metformin^a^		-	6 (5)	-
Anthropometry	Mean ± SD			
BMI (kg/m^2^), (*n* = 116^b^)		23.6 ± 5.4	21.5 ± 3.3	t = 4.1, *p* < 0.001
Waist circumference (cm), (*n* = 97^b^)		-	78.8 ± 9.9	-
Systolic blood pressure (mmHg), (*n* = 114^b^)		120.7 ± 11.7	120.4 ± 12.9	t = 0.30, *p* = 0.803
Diastolic blood pressure (mmHg), (*n* = 114^b^)		-	74.5 ± 9.1	-
Biochemistry	Mean ± SD			
HDL (*n* = 47^b^)		1.9 ± 0.6	1.5 ± 0.3	t = 7.0, *p* < 0.001
Triglycerides (*n* = 65^b^)		1.4 ± 1.1	0.9 ± 0.6	t = 4.8, *p* < 0.001
Fasting glucose (mmol/L) (*n* = 50^b^)		5.2 ± 1.3	5.0 ± 0.7	t = 1.7. p = 0.107

BMI: Body mass index, HDL: High-density lipoprotein, SD: Standard deviation.

^a^prescribed at any time during follow-up

^b^analytical sample.

^c^Analysis of means was conducted using an unpaired *t* test. Analysis of proportions was conducted using the chi-square equality of proportions test.

### Primary external validation analysis

**Figure 1.**
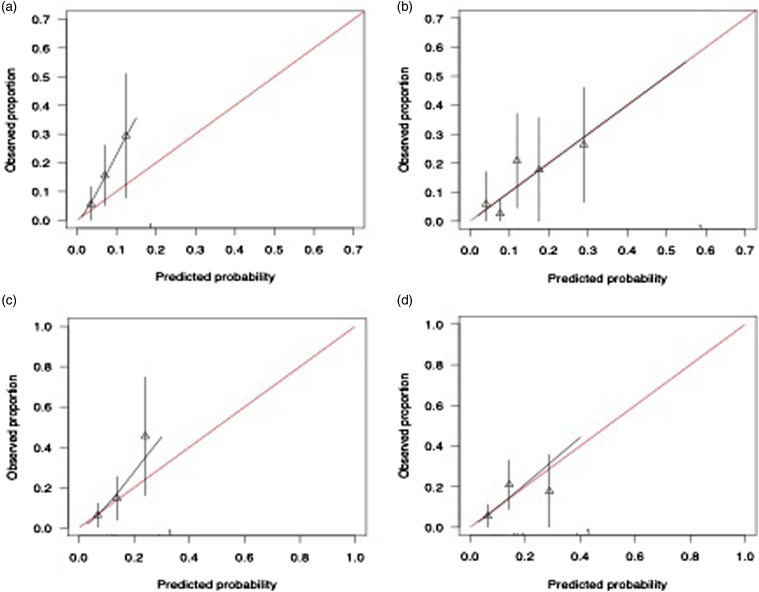
Calibration plots of PsyMetRiC in the Australian sample. A = full model before logistic calibration (primary analysis); B = full model after logistic calibration; C = partial model before logistic calibration (primary analysis); D = Partial model after logistic calibration. Calibration plots illustrate agreement between the observed (*y* axis) and predicted risk (*x* axis). Perfect agreement would trace the red line. Algorithm calibration is illustrated by the black line. Triangles denote grouped observations for participants at deciles of predicted risk, with 95% C.I.’s indicated by the vertical black lines. ^a^Logistic calibration takes into account differences in baseline risk that may exist between populations by re-estimating the intercept term, and also re-estimates the slope term thus assuming similar *relative* effects of the predictors but allowing for a larger or smaller *absolute* effect of the predictors. See Methods.

Numerical results for the full- and partial models are presented in Table 2. Calibration plots are presented in Figures 1 and S6. In summary, C-statistics for both PsyMetRiC versions were acceptable (Full model C = 0.72, 95% C.I., 0.65, 0.77; Partial model C = 0.71, 95% C.I., 0.64-0.75). Calibration plots were similar across imputed datasets, showing consistent under-prediction of risk, more extreme for the full model, with predictions becoming more inaccurate at higher predicted risk scores.

**Table 2.** Predictive performance statistics of the PsyMetRiC full- and partial models before and after updating and recalibration.

Measure of predictive performance	Primary Analysis, estimate (95% C.I.)		After updating and Calibration, estimate (95% C.I.)	
	Full-model	Partial-model	Full-model	Partial-model
C-statistic	0.72 (0.65, 0.77)	0.71 (0.64, 0.75)	0.74 (0.67, 0.80)	0.72 (0.65, 0.78)
r^2^	0.125 (0.08, 0.18)	0.109 (0.06, 0.17)	0.177 (0.14, 0.21)	0.146 (0.11, 0.18)
Calibration intercept	0.362 (0.19, 0.56)	0.199 (0.09, 0.32)	0.009 (−0.993, 0.01)	0.014 (−0.97, 0.03)
Calibration slope	1.187 (1.07, 1.30)	1.409 (1.27, 1.54)	1.003 (1.00, 1.01)	1.041 (1.01, 1.07)
Brier score	0.062 (0.04, 0.09)	0.069 (0.04, 0.11)	0.042 (0.03, 0.07)	0.047 (0.04, 0.06)

The C-statistic is a measure of discrimination and estimates the probability that a randomly selected ‘case’ will have a higher predicted probability than a randomly selected non-case. Scores of 1.0 indicate perfect discrimination; scores of >0.70 are generally considered acceptable. The calibration intercept (ideally close to 0) and calibration slope (ideally close to 1) are estimates of model calibration (i.e. the agreement between the observed proportion and predicted risk). The Brier score (ideally close to 0, with scores >0.25 indicating poor performance) is an overall measure of algorithm performance. For comparison, results from the original PsyMetRiC external validation in the UK were: full-model: C=0.75 (95% C.I., 0.69–0.80; r2 = 0.21 (95% CI., 0.18–0.25); Brier score=0.07 (95% C.I., 0.04–0.10); intercept=−0.05 (95% C.I., −0.08, −0.02); partial-model: C=0.74 (95% C.I., 0.67–0.79); r2=0.17 (95% C.I., 0.14–0.20); Brier score=0.08 (95% C.I., 0.05–0.11); intercept=−0·07 (95% C.I., −0.11, −0.03). See the original PsyMetRiC manuscript for further details^6^.

### Algorithm updating and recalibration

Numerical results for the full- and partial models post algorithm updating and recalibration are presented in Table 2. Calibration plots are presented in Figure 1 and Figure S7. In summary, C-statistics for both PsyMetRiC versions improved slightly (full model C = 0.74, 95% C.I., 0.67-0.80; partial model C = 0.72, 95% C.I., 0.65-0.78). Calibration plots were similar across imputed datasets, showing greatly improved agreement between predicted risk and observed proportions for both PsyMetRiC versions, though minor under-prediction of risk remained apparent for the partial model specifically.

### Clinical usefulness

**Figure 2.**
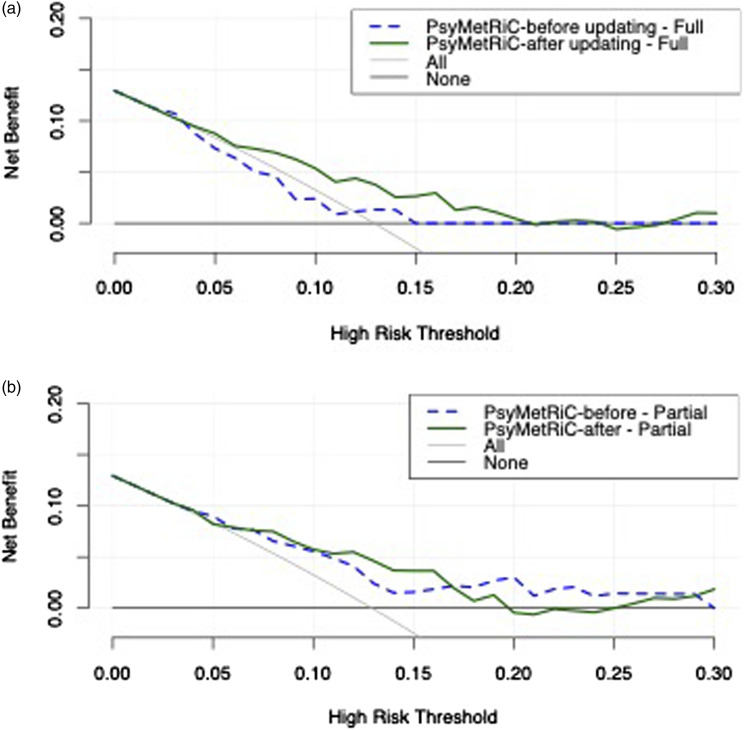
Clinical usefulness of PsyMetRiC in the Australian sample before and after logistic calibration. A = full-model; B = partial-model. The plot reports net benefit (*y* axis) of PsyMetRiC Full- and Partial-Models (blue line = original PsyMetRiC algorithm applied to the sample; red line = recalibrated site-specific version) across a range of risk thresholds (*x* axis) compared with intervening in all (grey line) or intervening in none (black line). In decision curve analysis, it is customary to consider only the range of risk thresholds that may reasonably be considered in clinical practice. Our upper-bound of 0.30 represents around a one-in-three chance of developing metabolic syndrome should nothing change, and it is unlikely that risk thresholds greater would be tolerated. Net harm (i.e. more false positives than true positives exposed to an intervention at a selected risk threshold) is indicated when the decision curve line is plotted at y < 0.

Decision curve analysis results are presented in Figure 2, Figure S8 and Tables S7-S8. In summary, results were broadly similar across imputed datasets and indicated a very narrow window for clinical usefulness using the original UK PsyMetRiC versions in Australia, across the studied risk thresholds, compared with competing strategies of intervening in all or none. However, after updating and recalibration, both PsyMetRiC versions showed much stronger potential for clinical usefulness in Australia across risk thresholds 5%–20%, compared with competing strategies of intervening in all or none (Figure 2, Figure S8). For example, at a risk threshold of 15%, the recalibrated full and partial PsyMetRiC versions provided net benefits of 0.05 (95% C.I., 0.00-0.10) and 0.04 (95% C.I., −0.02-0.08), respectively, meaning that an additional 36% of metabolic syndrome cases could be prevented with the full model, and 28% with the partial-model, with no increase in false positives (Tables S7-S8).

## Discussion

This study provides preliminary evidence that the PsyMetRiC cardiometabolic risk prediction algorithm, originally developed and validated in the UK,^7^ may be clinically useful for young people with psychotic disorders in Australia. The discrimination performance, as measured by the C-statistic, was acceptable (>0.7) in both the full- and partial models, with agreement between observed and predicted risk improving with logistic calibration.

The recalibrated algorithms for full- and partial models in Australia (C = 0.74; C = 0.72) performed similar to the original study in the UK (C = 0.75; C = 0.74),^7^ and the follow-up study in Swiss (C = 0.73; C = 0.68) and Spanish samples (C = 0.72; C = 0.66).^8^ These findings indicate the recalibrated model assigns a higher risk value to someone who develops metabolic syndrome, than someone who doesn’t, approximately three-quarters of the time. Our results indicate that PsyMetRiC is likely to be clinically useful for interventions that could be recommended for risk thresholds falling between 5 and 20%. For example, such interventions may include enrolment into a lifestyle program (where relatively low risk thresholds may be tolerated before the benefits of the intervention are likely to outweigh the risks), or the prescription of metformin (where slightly higher risk thresholds may be tolerated, due to the potential for adverse effects and/or increased medication burden).

### Limitations

These results are preliminary given the sample size of 116 people from a single health district in Australia and an outcome prevalence of 13%, which was slightly lower than the original PsyMetRiC study. In the generation of the site-specific recalibrated version of PsyMetRiC, data from the whole sample was used to generate recalibration estimates. Therefore, further external validation is required in future to confirm generalisability within other samples. The dataset also contained considerable missing data, particularly for blood results; however, we were able to partially address this limitation through detailed missing sample analysis and multiple imputation. The inclusion of certain variables within the model was limited, for example, ethnicity was unavailable and instead the variable ‘Born in Australia’/‘Born Outside of Australia’ was included. In addition, the number of people who identified as Aboriginal and/or Torres Strait Islander in this dataset was limited to *n* = 3. Recalibration through a larger dataset should allow for Aboriginal and/or Torres Strait Islander status as a predictor variable given the known difference in health outcomes. In the Swiss and Spanish samples^8^ discrimination and calibration in people with prior antipsychotic exposure were similar to the main analytic samples. However, we were unable to determine whether the site-specific algorithm performs at an acceptable level for people who are not antipsychotic naïve. Finally, while we matched the original validation study (UK) for age parameters for inclusion, the mean age was lower in our sample, largely due to the majority of sample coming from a targeted Early Psychosis Programme for 15-25-year-olds.

### Future directions

While PsyMetRiC is not yet ready for routine clinical use in Australia, there is a clear path ahead to achieve that milestone in the future. PsyMetRiC now requires testing in larger Australian datasets, with further logistic calibration and updating as necessary. This may further improve calibration, may permit the modelling of individual antipsychotics to improve discrimination, longer timeframes for outcome prediction, an ability to predict outcome severity, and an ability to predict distal cardiometabolic diseases such as type-2 diabetes and cardiovascular disease. Engagement with stakeholders locally is also required for the development of cut-off thresholds for specific interventions.

If the Australian versions of PsyMetRiC demonstrate adequate accuracy and net benefit in a population-level sample, implementation studies would aid in determining an appropriate implementation and delivery strategy within routine clinical care. The resulting application and implementation strategy could be used within clinical practice in Australian mental health services as a tool for informed and shared decision-making,^14^ and facilitate engagement with additional strategies, such as lifestyle intervention, to reach targets set-out in the Healthy Active Lives (HeAL) international declaration (https://www.iphys.org).^15^

## Conclusion

An updated and recalibrated PsyMetRiC model for the Australian population shows promise. Validation in a larger sample size is required to confirm its accuracy and clinical usefulness before routine clinical use in Australia.

## Supplemental Material


Supplemental Material - Metabolic syndrome risk prediction in an Australian sample with first-episode psychosis using the psychosis metabolic risk calculator: A validation study
Supplemental Material for Metabolic syndrome risk prediction in an Australian sample with first-episode psychosis using the psychosis metabolic risk calculator: A validation study by Scott B Teasdale, Oliver Ardill-Young, Rachel Morell, Philip B Ward, Golam M Khandaker, Rachel Upthegrove, Jackie Curtis and Benjamin I Perry in Australasian Psychiatry.

## Data Availability

Participant data cannot be publicly deposited due to patient and participant confidentiality. Participant data may be accessed after formal application to, and approval by, the South Eastern Sydney Local Health District Human Research Ethics Committee. This study did not receive any specific grant funding. Data were collected as part of routine clinical care. Data were extracted and analysed as part of the authors’ academic roles.
