# Supplementary material for: Metabolic syndrome risk prediction in an Australian sample with first-episode psychosis using the psychosis metabolic risk calculator: A validation study
Source: Australas Psychiatry. 2024 Aug 13;33(1):120–7. doi: 10.1177/10398562241269171 (PMC11804134; doi:10.1177/10398562241269171)
Supplement: Supplemental Material - Metabolic syndrome risk prediction in an Australian sample with first-episode psychosis using the psychosis metabolic risk calculator: A validation study [file sj-pdf-1-apy-10.1177_10398562241269171.pdf]

Metabolic syndrome risk prediction in an Australian sample with first-episode psychosis using the Psychosis Metabolic Risk Calculator: a Validation study.

Supplementary Data

Part A - Supplementary Methods

Procedure for Collecting Anthropometry, Biochemistry and Blood Pressure.

Body weight was measured by OMRON HN-283 digital scales to the nearest 0.1kg, with the client barefoot and wearing light clothing. Height was measured using a stadiometer to the nearest 1cm. BMI was calculated as weight (kg)/height^2 (m). Waist circumference was measured horizontally at the navel at the end of expiration to the nearest 0.1cm. Waist circumference was categorised as 'at risk' according to ethnic specific values from the IDF criteria: Europids (≥80cm for females and ≥94cm for males) and Asian people (≥80cm for females and ≥90cm for males).[1]

Blood pressure was measured using a sphygmomanometer with the client in a seated position and reported as millimetres of mercury (mmHg). Normal blood pressure reference ranges were: systolic blood pressure <130mmHg, and diastolic blood pressure <85mmHg.[1] Metabolic bloods (lipids and glucose) were collected and analysed following a 10-hour fast by a pathology service and reported as millimoles per litre (mmol/L). In the event of a non-fasting test, blood glucose was reported as random. Normal reference ranges for metabolic biochemistry were: total cholesterol <5.6mmol/L, LDL ≤4.0 mmol/L, HDL ≥1.03 men and ≥1.29 women, triglycerides <1.7mmol/L, fasting glucose <5.6mmol/L, and random glucose <7mmol/L.[1]

Table S1: Original PsyMetRiC algorithm (UK) coefficients after shrinkage for optimism

PsyMetRiC Predictor	Full-Model	Partial-Model

Intercept	-6.439813	-6.973829
Age in years (continuous)	0.006233226	0.00633115
Black/African-Caribbean Ethnicity (yes/no)	0.004258861	0.07548129
Asian / Other Ethnicity (yes/no)	0.211217746	0.29285950
Male Sex (yes/no)	0.222300765	0.31460036
Body Mass Index (BMI) (kg/m^2) (continuous)	0.141186241	0.16912161
Current Smoking Status (smoker, non-smoker)	0.153691193	0.24751854
Prescribed a Metabolically-Active Antipsychotic[a] (yes/no)	0.497552758	0.60013558
High-Density Lipoprotein (HDL) (mmol/L) (continuous)	-0.399013329	-
Triglycerides (mmol/L) (continuous)	0.343528440	-

Definition of More or Less Metabolically-Active Antipsychotic Medications

In the original PsyMetRiC development study,[2] antipsychotic medication prescription was reduced into a binary variable representing more or less metabolically-active medications, guided by meta-analytic evidence. See Table S2 for the classification and evidence used to make the classification.

Table S2: Classification of More Metabolically-Active Antipsychotics

More Metabolically-Active Antipsychotics	Less Metabolically-Active Antipsychotics
Olanzapine[3]	Aripiprazole[3]
Quetiapine[3]	Amisulpride[3]
Risperidone[3]	Haloperidol[3]
Paliperidone[3]	Sulpiride[4]
Clozapine[3]	Pericyazine[5]
Chlorpromazine[3]	Lurasidone[3]
Asenapine[6]	Ziprasdone[3]
Pimozide[4]	Flupenthixol[6]
Levomepromazine[4]	Fluphenazine[6]
Prochlorperazine[6]	Zuclopenthixol[4]
Trifluoperazine[7]	
Pipotiazine[7]	

This table comprises all antipsychotics prescribed for participants/patients in the original PsyMetRiC development study.[2]

Missing Data

The amount and structure of missing data in the sample was assessed, and considered for multiple imputation using chained equations.[8] To examine the pattern of missing data in the sample, we

plotted a ‘missingness map’ (Figure S1) and matrix comparing missing values in predictor variables against each other (Figure S2). The missingness map below shows a matrix of paired variables to show relationships between observed data (light blue) and missing data (grey) between the following variables at both baseline and follow-up: age; sex; BMI; waist circumference; smoking status; metabolically-active antipsychotic; mood stabiliser; metformin; high-density lipoprotein cholesterol; triglycerides; metabolic syndrome; and engagement in lifestyle intervention (Keeping the Body In Mind [KBIM]).

Figure S1: Missingness Map

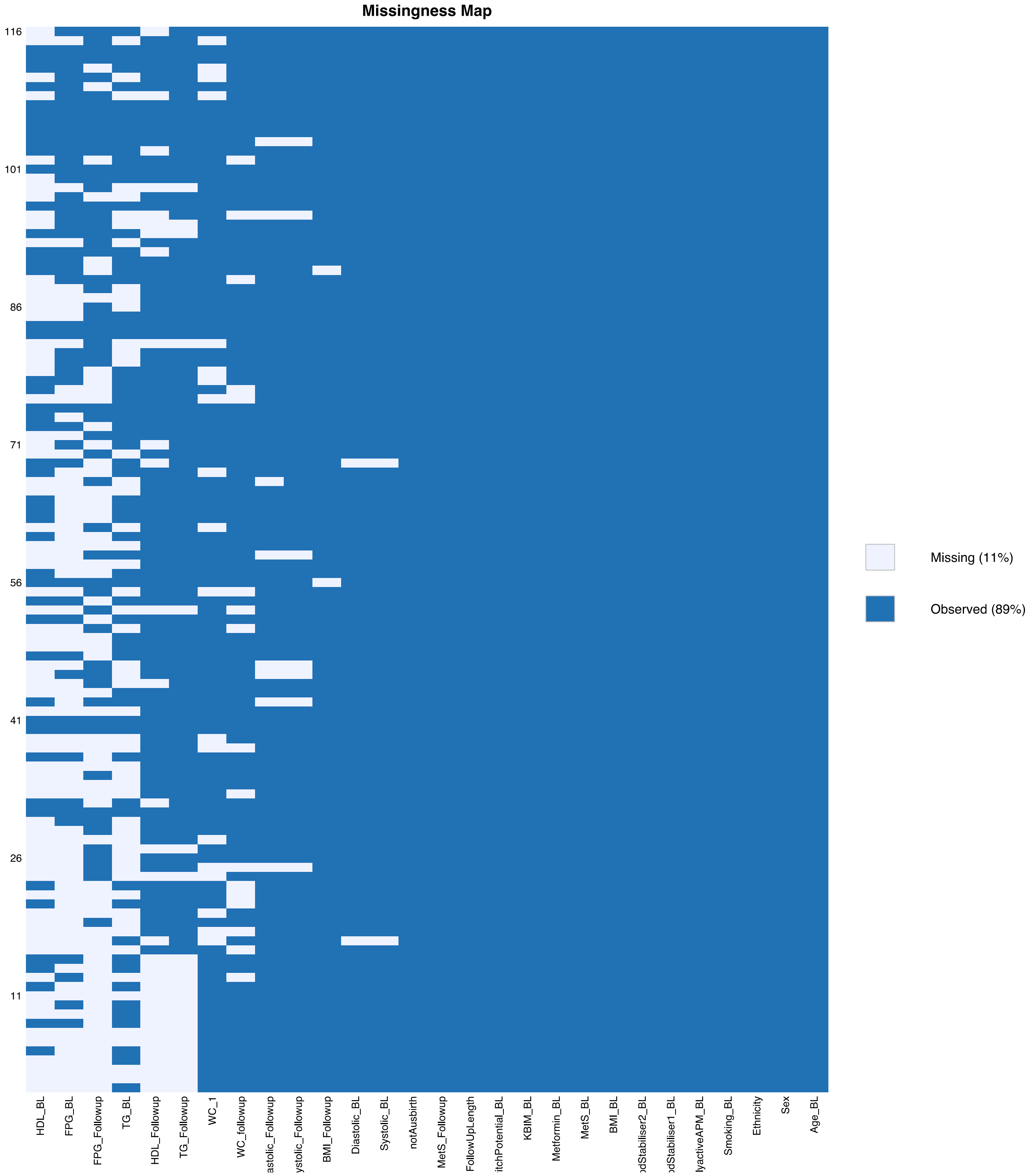

Missingness maps help to visualize patterns in missing data (white bars) compared with observed data (blue bars) between variables. BL: baseline, BMI: body mass index, FPG: fasting plasma glucose, HDL: high density lipoprotein, KBIM: Keeping the Body In Mind Lifestyle Program, MetS: metabolic syndrome, TG: triglycerides, WC: waist circumference.

Figure S2: Matrix of Missing Values Per Predictor Variable

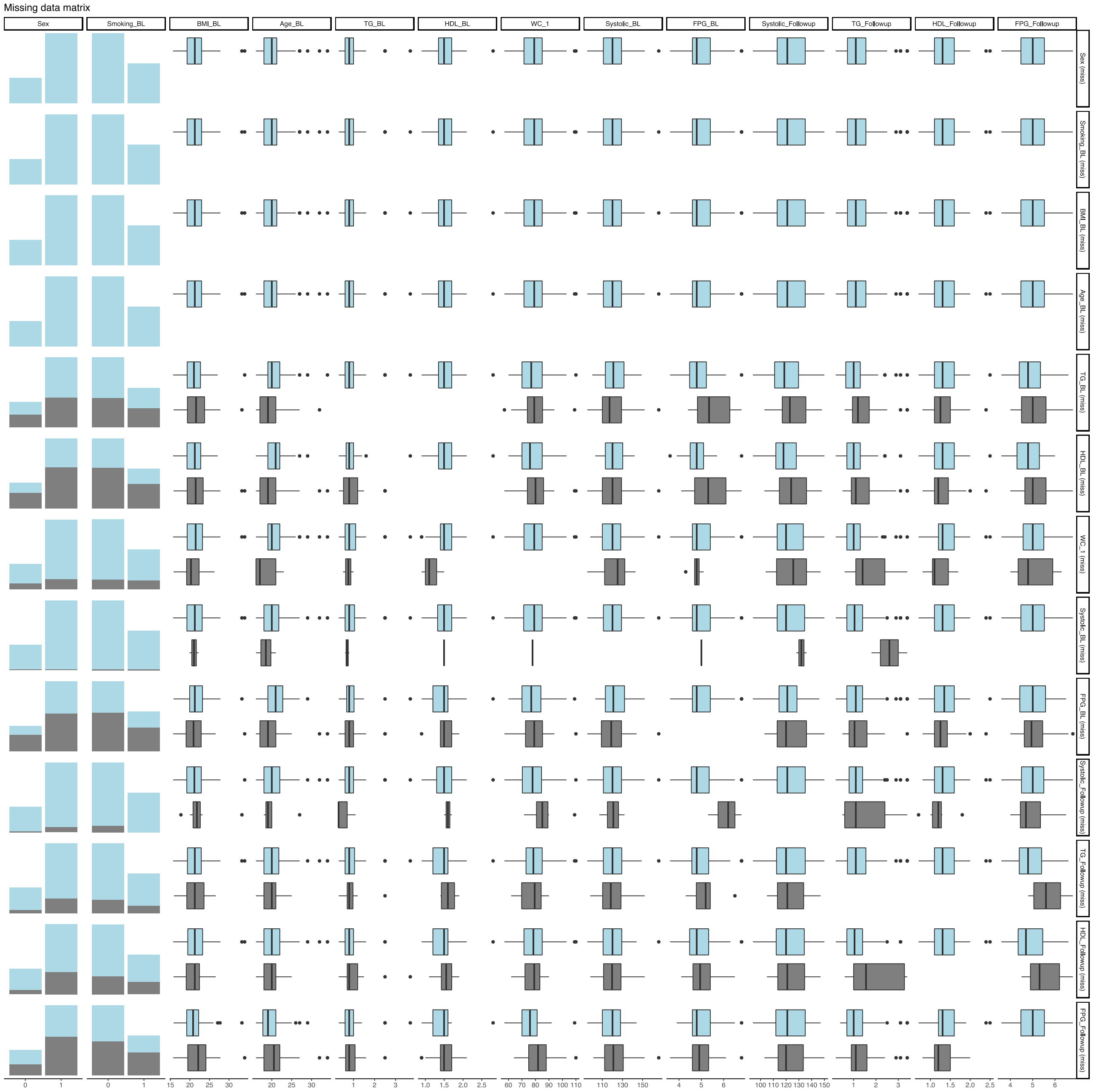

From the missingness map plot (Fig S1), there was a weak pattern of missing data such that when biochemical tests were not performed, all biochemical variables were missing together, as would be expected. However, this missingness pattern was not present in all individuals. There were no other common patterns of missing data between variables. From the matrix of missing values (Fig S2), there is evidence of an association between sex and missingness for biochemical data, with females more likely to have missing biochemical data; and an association between smoking status and missingness for biochemical data, with non-smokers more likely to have missing biochemical data. Therefore, because some patterns of missing data were evident in our sample, we only considered

variables for imputation where they were <50% missing[9] and had suitable auxiliary variables available for use (metformin prescription, changes to psychotropic medication prescription during follow-up period, and engagement with lifestyle intervention during follow-up period) as indicators of missingness, in order to reduce the impact of 'missing not at random' bias.[10] See Table S3 for the proportion of missing data for each variable. We imputed 10 datasets in both samples. Box-and-Whisker (Fig S3) and Density plots (Fig S4) were used to check similarities of observed and imputed data. The MetS outcome was calculated from the constituent variables after the multiple imputation step.

Table S3. Proportion of Missing Data Per Variable

**Variable**	**Australia**
Sex	0.00
Ethnicity	0.00
Age	0.00
Smoking Status	0.00
Antipsychotic Prescription	0.00
SBP – Baseline	0.02
SBP – Follow-up	0.06
BMI – Baseline	0.00
BMI – Follow-up	0.02
Triglycerides – Baseline	0.34
Triglycerides – Follow-up	0.19
HDL – Baseline	0.49
HDL – Follow-up	0.28

SBP = systolic blood pressure; BMI = body mass index; HDL = High-density lipoprotein cholesterol.

For numerical-based analyses, estimates were pooled using Rubin's rules. For plot-based analyses, plots were generated in each imputed dataset and checked for similarity, with one randomly selected plot per analysis presented in the main manuscript and all remaining plots presented in the Supplementary Data.

Figure S3: Box and Whisker Plot to Check Imputed Data

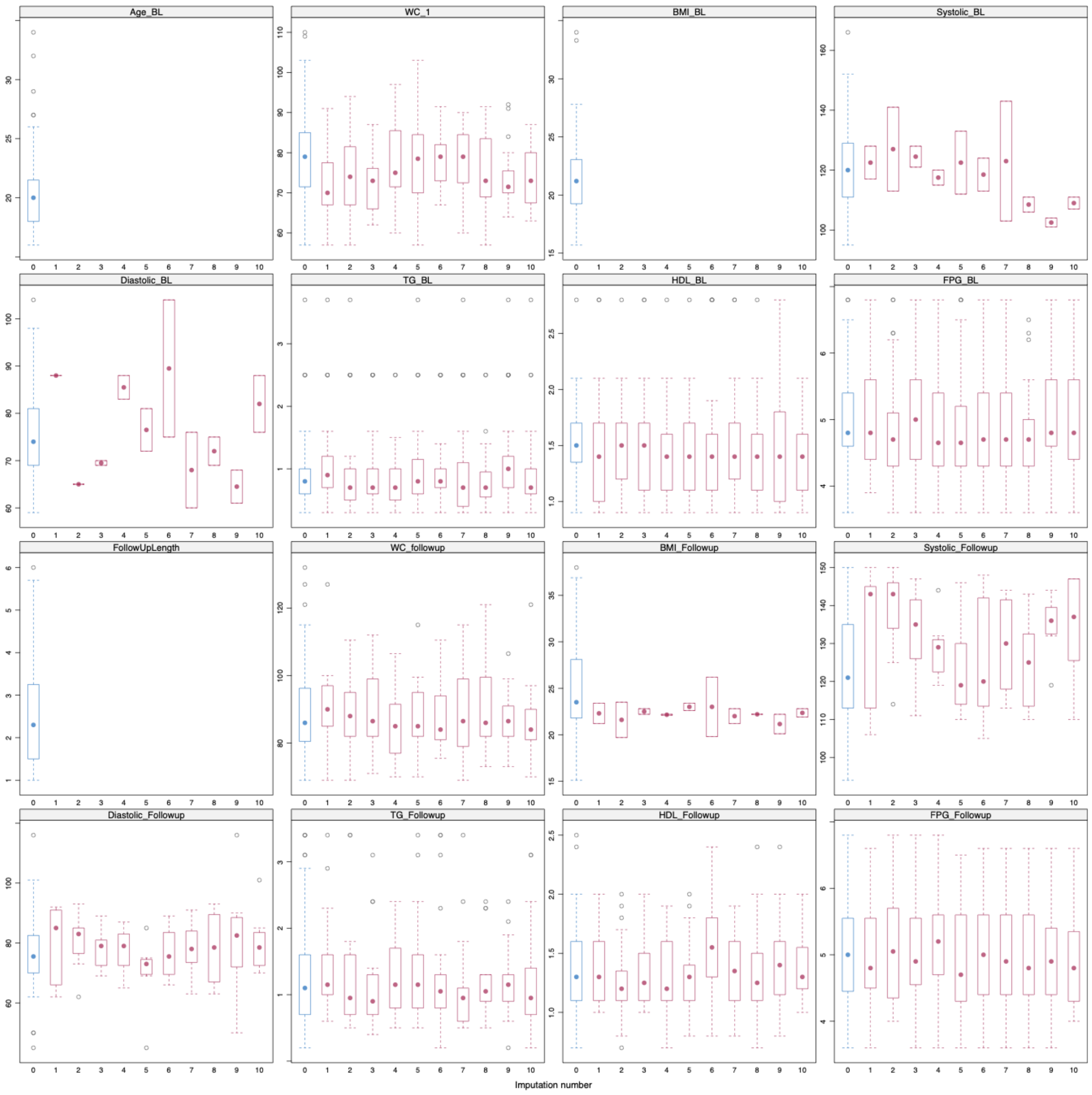

The box and whisker plots display the distribution of observed (blue) and imputed (pink) data for each imputed variable, across 10 imputed datasets.

Figure S4: Strip Plot to Check Imputed Data

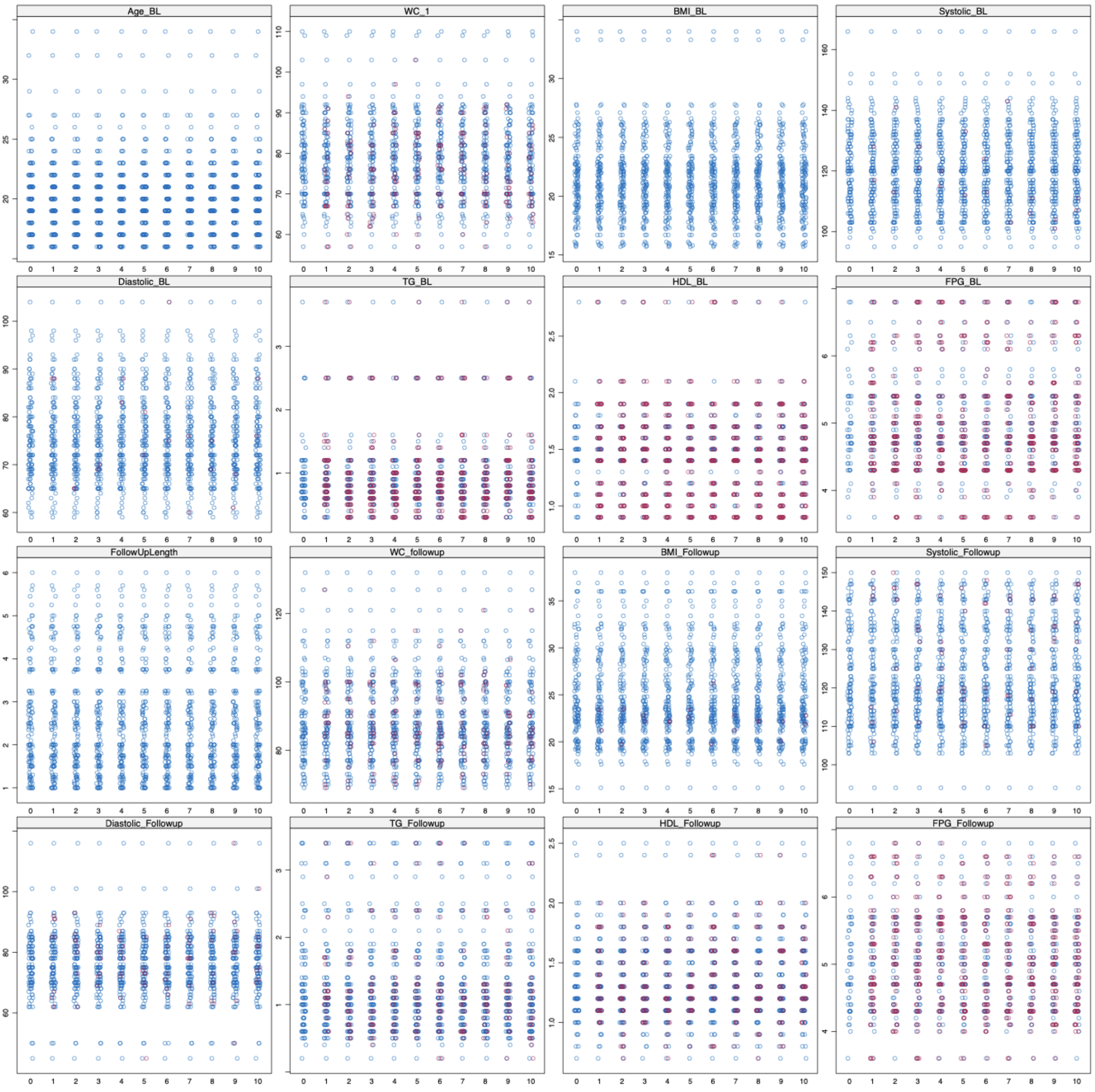

These one-dimensional scatterplots show the observed (blue) and imputed (pink) data points, for each imputed variable across 10 imputed datasets.

External Validation Analysis

The C-statistic is derived from the area under the curve and estimates the probability that a randomly selected 'case' (participant who develops the outcome) will have a higher predicted probability for incident MetS than a randomly selected 'non-case' (participant who does not develop the outcome). Scores of 1.0 indicate perfect discrimination; scores of 0.5 indicate that the algorithm is no better than chance; scores of >0.7 are generally considered acceptable.[11] Calibration

plots estimate the accuracy of absolute-risk estimates (i.e. agreement between observed and predicted risk).

Model Updating with Additional Variable and Recalibration

PsyMetRiC was updated by adding a new variable (non-Australian birth), as the closest available proxy in the available dataset to the 'ethnicity' variable of PsyMetRiC, which was coded based on the ethnic makeup of the UK population and is thus not appropriate for the Australian population. To do this, we fit a logistic regression model in the sample with the linear predictor from the primary external validation analysis alongside the new variable as the sole two predictors in a logistic regression model. After extracting averaged intercept and slope coefficients for the recalibrated model, heuristic shrinkage[12] was applied to account for over-optimism of the newly estimated variable. The heuristic shrinkage factor is defined as (model χ^2 – *df* / model χ^2), where model χ^2 refers to the difference in -2 log-likelihood between a model with and without predictors, and *df* refers to the degrees of freedom used by the predictors.[12] The individual linear predictors and predicted probabilities for each participant were then recalculated using the updated intercept, slope and new variable coefficients and predictive performance was re-assessed.

Decision Curve Analysis: Clinical Usefulness and Potential Cut-offs

Decision curve analysis[13] was used to assess the clinical usefulness of PsyMetRiC by estimating net benefit (Supplementary Methods). Net benefit is a metric of true positives minus false positives, and is calculated as: sensitivity × prevalence – (1 – specificity) × (1 – prevalence) × *w*,

where *w* is the outcome odds at a given risk threshold.[14] The risk threshold is the amount of tolerable risk before an intervention is deemed necessary. Net benefit incorporates the consequences of the decisions made on the basis of an algorithm, and is therefore preferable to related measures

such as sensitivity and specificity alone.[14] We also reported the standardized net benefit (net benefit / outcome prevalence) and related metrics (sensitivity and specificity) across a range of reasonable risk thresholds. We drew a decision curve plot which visualised and compared the net benefit of the original PsyMetRiC algorithm vs the recalibrated country-specific version in each respective sample, compared with intervening in all, or intervening in none. Classical decision theory proposes that at a chosen risk-threshold, the choice with the greatest net-benefit should be preferred.[14]

Sample Size Power Calculation

We used recently developed criteria[15,16] to estimate the recommended sample size for our analysis. Since our study features retrospectively collected data with the available sample fixed, we used these calculations as a means to estimate the likely precision of our analysis. Briefly, the calculation[8] for external validation is performed based on the anticipated outcome prevalence, the anticipated C-statistic, and the required standard errors (SEs) of the estimated C-statistic, calibration slope, and calibration-in-the-large. The calculation[9] for developing a model with n=1 additional predictors is based on the anticipated outcome prevalence, the anticipated C-statistic, and the anticipated shrinkage of the predictor required to account for likely over-fitting. We obtained the anticipated outcome prevalence (16.86%) and C-statistic (0.75) from the external validation of the original PsyMetRiC study. The shrinkage parameter was set as recommended to 0.9.[16]

Based on the available sample, the expected SE for the C-statistic was 0.06. The expected SEs for the calibration slope and calibration-in-the-large were 0.31 & 0.27 respectively. For reference, for more precise[15] SEs of the C-statistic (0.025), calibration slope (0.10) and calibration-in-the-large (0.10), a minimum sample size of n=1,052 would be required.

The recommended sample size for developing a model with n=1 additional predictors is n=216.

Therefore, results from both the primary external validation analysis and model updating procedure should be regarded as preliminary, with further validation analysis conducted in larger sample sizes of the Australian population required.

Table S4. Analysis of Multi-Collinearity of Variables Included in PsyMetRiC in Australian Sample

Predictor	Variance Inflation Factor[a]
Sex	2.40
Age	2.02
Body Mass Index	1.56
Current Smoking Status	3.17
Prescribed a More Metabolically-Active Antipsychotic	1.00
Triglycerides	3.67
HDL Cholesterol	1.80

[a]Scores close to 1 indicate no evidence of multi-collinearity. Scores >5 indicate potential multi-collinearity.

Part B: Supplementary Results Tables

Table S5: Key Comparison Metrics Between External Validation Sample and PsyMetRiC Development Sample

Category	***Comparator***	***Country***	
		**United Kingdom**	**Australia**
**General**	Area (km^2)	243,610	7,741,220
	Government Form	Parliamentary Constitutional Monarchy	Parliamentary Constitutional Monarchy
**Demographics**	Population, millions	67.22	26.01
	Inhabitants/km^2	275.92	3.4
	Life Expectancy (Males) (years)	79	81.3
	Life Expectancy (Females) (years)	83	85.4
	Quality of Life Score[1] – Political Stability[2]	75	89
	Quality of Life Score[1] – Civil Rights[3]	91	94
	Quality of Life Score[1] – Health[4]	78	87
	Quality of Life Score[1] – Cost of Living[5]	34	31
	Prevalence of Smoking (%)[6]	15.4	10.1
	Dominant Language (language, %)	English (97)	English (77)
	Dominant Religion (religion, %)	Christianity (67)	Christianity (44)
	Ethnic Fractionalization Score[7]	0.12	0.09
	Linguistic Fractionalization Score[7]	0.05	0.33
	Religious Fractionalization Score[7]	0.69	0.82
	Annual Net Migration per 1000	3.9	5.2

	Inhabitants[8]		
**Economy**	Unemployment Rate (%)	4.5	3.7
	Median income (USD)	39,830	53,040
	Corruption Index[9]	78	75
**Healthcare**	Hospital Beds per 1000	2.46	3.80
	Health Expenditure (%GDP)[10]	9.8	10.6
	Psychiatric Inpatient Beds per 1000[11]	0.46	0.03
	Medical Doctors per 1000	2.81	4.10

Information obtained from *worlddata.info* unless otherwise stated. [1]scored from 0 (worst) – 100 (best). See https://www.worlddata.info/quality-of-life.php for more information on how scores were created. [2]A metric comprising economic inflation, government debt/deficits, gross domestic product, unemployment rate, and ratio of available money supply to currency reserves. [3]A metric comprising the regulatory quality of the government, democratic participation by populus, and the corruption index from *Transparency.org*. [4]A metric comprising average life expectancy, drinking water supply, and the number of medical doctors and hospital beds. [5]A metric comprising cost of living, median income, government taxation, and The World Bank 'Ease of Doing Business' index. [6]Data obtained via *The World Bank* https://data.worldbank.org; [7]Fractionalization scores are commonly used metrics in economics, and show the probability that two randomly drawn individuals from the population are not from the same group (e.g., ethnic, religious, linguistic). 2023 fractionalization scores were taken from *worldpopulationreview.com*. [8]Data obtained from United Nations https://population.un.org/. [9]based on scores from *Transparency International* Corruption Perceptions Index (https://www.transparency.org/en/cpi/2021/index/). [10]Taken from OECD Health At a Glance. [11]Taken from World Health Organization European Health Information Gateway & OECD.

Table S6: Results of Logistic Calibration of PsyMetRiC in Australian Sample

PsyMetRiC Version	New Intercept	New Slope	Coefficient for Non-Australian Birth[1]
Full Model	-5.709677348	1.196114641	-0.393403214
Partial Model	-6.775837389	1.165877769	-0.349797536

[1]Coefficient shrunk for optimism using heuristic shrinkage[12]. See Methods and Supplementary Methods.

Table S7: Pooled Net Benefit and Standardized Net Benefit of The PsyMetRiC Full-Model (Original and Recalibrated Versions) Across a Range of Feasible Risk Thresholds

Risk Threshold[a]	Net Benefit (Recalibrated) + 95% CI	Net Benefit (Original) + 95% CI	Standardized Net Benefit (Recalibrated) + 95% CI[b]	Standardized Net Benefit (Original) + 95% CI[b]
0.00	0.129 (0.069,0.198)	0.129 (0.06,0.19)	1 (1.000,1.000)	1 (1.000,1.000)
0.01	0.121 (0.06,0.19)	0.121 (0.051,0.181)	0.932 (0.864,0.959)	0.932 (0.843,0.957)
0.02	0.112 (0.05,0.182)	0.112 (0.042,0.173)	0.864 (0.73,0.917)	0.867 (0.7,0.914)
0.03	0.095 (0.034,0.157)	0.098 (0.036,0.151)	0.735 (0.485,0.855)	0.758 (0.466,0.87)
0.04	0.09 (0.03,0.153)	0.096 (0.031,0.15)	0.697 (0.417,0.833)	0.742 (0.462,0.858)
0.05	0.085 (0.025,0.148)	0.088 (0.032,0.143)	0.66 (0.342,0.808)	0.681 (0.401,0.836)
0.06	0.081 (0.021,0.145)	0.064 (0.016,0.117)	0.627 (0.296,0.784)	0.496 (0.217,0.72)
0.07	0.08 (0.02,0.144)	0.059 (0.015,0.106)	0.622 (0.268,0.774)	0.454 (0.194,0.677)
0.08	0.07 (0.015,0.136)	0.061 (0.019,0.109)	0.542 (0.188,0.749)	0.472 (0.215,0.703)
0.09	0.069 (0.011,0.133)	0.037 (0.007,0.078)	0.53 (0.14,0.74)	0.288 (0.062,0.514)

0.10	0.072 (0.015,0.135)	0.039 (0.008,0.078)	0.556 (0.183,0.753)	0.304 (0.063,0.511)
0.11	0.053 (0.0,0.105)	0.044 (0.013,0.081)	0.412 (-0.006,0.634)	0.342 (0.126,0.575)
0.12	0.055 (0.002,0.107)	0.029 (0.0,0.061)	0.424 (0.027,0.652)	0.221 (0.003,0.449)
0.13	0.046 (-0.005,0.097)	0.021 (-0.005,0.05)	0.358 (-0.076,0.601)	0.16 (-0.046,0.4)
0.14	0.047 (-0.002,0.1)	0.023 (-0.001,0.052)	0.363 (-0.021,0.618)	0.178 (-0.012,0.423)
0.15	0.047 (-0.003,0.099)	0 (0,0)	0.361 (-0.029,0.634)	0 (0,0)
0.16	0.04 (-0.005,0.095)	0 (0,0)	0.308 (-0.058,0.583)	0 (0,0)
0.17	0.039 (-0.006,0.094)	0 (0,0)	0.3 (-0.082,0.584)	0 (0,0)
0.18	0.042 (-0.004,0.098)	0 (0,0)	0.322 (-0.044,0.595)	0 (0,0)
0.19	0.043 (-0.005,0.096)	0 (0,0)	0.334 (-0.063,0.61)	0 (0,0)
0.20	0.026 (-0.017,0.08)	0 (0,0)	0.2 (-0.222,0.5)	0 (0,0)
0.21	0.0 (-0.034,0.044)	0 (0,0)	0.001 (-0.35,0.27)	0 (0,0)
0.22	-0.011 (-0.046,0.031)	0 (0,0)	-0.082 (-0.464,0.191)	0 (0,0)
0.23	-0.01 (-0.045,0.032)	0 (0,0)	-0.079 (-0.468,0.194)	0 (0,0)
0.24	-0.007 (-0.04,0.036)	0 (0,0)	-0.053 (-0.364,0.219)	0 (0,0)
0.25	-0.006 (-0.037,0.034)	0 (0,0)	-0.044 (-0.361,0.231)	0 (0,0)
0.26	-0.001 (-0.033,0.042)	0 (0,0)	-0.011 (-0.324,0.264)	0 (0,0)
0.27	0.004 (-0.026,0.047)	0 (0,0)	0.027 (-0.256,0.311)	0 (0,0)
0.28	0.009 (-0.02,0.052)	0 (0,0)	0.07(-0.181,0.319)	0 (0,0)
0.29	0.008 (-0.021,0.051)	0 (0,0)	0.064 (-0.191,0.314)	0 (0,0)
0.30	0.011 (-0.018,0.05)	0 (0,0)	0.086 (-0.165,0.33)	0 (0,0)

[a]Different risk thresholds may be selected depending on the proposed intervention (i.e., balancing the risk/benefit of exposing false positives to an intervention to benefit the most true positives), as well as patient or clinician preference.

[b]Standardised net benefit is calculated as the net benefit / outcome prevalence, showing the proportion of improvement in net benefit at the selected risk threshold.

Table S8: Pooled Net Benefit and Standardized Net Benefit of The PsyMetRiC Partial-Model (Original and Recalibrated Versions) Across a Range of Feasible Risk Thresholds

Risk Threshold[a]	Net Benefit (Recalibrated) + 95% CI	Net Benefit (Original) + 95% CI	Standardized Net Benefit (Recalibrated) + 95% CI[b]	Standardized Net Benefit (Original) + 95% CI[b]
0.00	0.129 (0.069 , 0.19)	0.129 (0.078 , 0.198)	1 (1 , 1)	1 (1 , 1)
0.01	0.121 (0.06 , 0.181)	0.121 (0.068 , 0.19)	0.932 (0.864 , 0.957)	0.932 (0.88 , 0.959)
0.02	0.112 (0.05 , 0.173)	0.112 (0.059 , 0.182)	0.863 (0.724 , 0.913)	0.863 (0.757 , 0.917)
0.03	0.103 (0.04 , 0.165)	0.103 (0.049 , 0.174)	0.794 (0.582 , 0.869)	0.794 (0.632 , 0.878)
0.04	0.096 (0.033 , 0.158)	0.095 (0.04 , 0.167)	0.742 (0.474 , 0.835)	0.733 (0.514 , 0.841)
0.05	0.082 (0.025 , 0.137)	0.09 (0.034 , 0.162)	0.635 (0.316 , 0.773)	0.695 (0.433 , 0.817)
0.06	0.079 (0.023 , 0.136)	0.078 (0.02 , 0.152)	0.61 (0.264 , 0.751)	0.601 (0.263 , 0.779)
0.07	0.076 (0.018 , 0.136)	0.077 (0.02 , 0.151)	0.587 (0.215 , 0.742)	0.592 (0.247 , 0.778)
0.08	0.075 (0.014 , 0.136)	0.066 (0.012 , 0.14)	0.58 (0.183 , 0.753)	0.507 (0.138 , 0.727)
0.09	0.065 (0.002 , 0.126)	0.061 (0.013 , 0.129)	0.504 (0.018 , 0.693)	0.47 (0.16 , 0.697)

0.10	0.057 (-0.006, 0.11)	0.056 (0.009, 0.118)	0.444 (-0.083, 0.644)	0.43 (0.062, 0.71)
0.11	0.053 (-0.01, 0.105)	0.049 (-0.005, 0.111)	0.412 (-0.103, 0.609)	0.378 (-0.058, 0.673)
0.12	0.055 (-0.009, 0.107)	0.041 (-0.004, 0.101)	0.424 (-0.095, 0.618)	0.318 (-0.042, 0.591)
0.13	0.046 (-0.012, 0.092)	0.024 (-0.02, 0.077)	0.358 (-0.153, 0.612)	0.188 (-0.193, 0.445)
0.14	0.037 (-0.014, 0.083)	0.015 (-0.021, 0.072)	0.285 (-0.186, 0.55)	0.116 (-0.208, 0.438)
0.15	0.037 (-0.016, 0.083)	0.016 (-0.021, 0.072)	0.282 (-0.209, 0.546)	0.122 (-0.193, 0.442)
0.16	0.037 (-0.014, 0.083)	0.018 (-0.019, 0.077)	0.283 (-0.185, 0.533)	0.143 (-0.165, 0.46)
0.17	0.02 (-0.029, 0.059)	0.022 (-0.014, 0.08)	0.153 (-0.329, 0.406)	0.169 (-0.128, 0.505)
0.18	0.007 (-0.033, 0.044)	0.02 (-0.016, 0.079)	0.055 (-0.436, 0.319)	0.158 (-0.15, 0.497)
0.19	0.013 (-0.026, 0.049)	0.027 (-0.007, 0.081)	0.099 (-0.33, 0.402)	0.208 (-0.074, 0.543)
0.20	-0.004 (-0.034, 0.028)	0.03 (-0.004, 0.084)	-0.033 (-0.455, 0.202)	0.233 (-0.036, 0.553)
0.21	-0.006 (-0.038, 0.027)	0.012 (-0.014, 0.051)	-0.048 (-0.483, 0.191)	0.094 (-0.155, 0.34)
0.22	-0.001 (-0.029, 0.031)	0.019 (-0.01, 0.058)	-0.007 (-0.333, 0.231)	0.144 (-0.088, 0.382)
0.23	-0.002 (-0.031, 0.029)	0.021 (-0.007, 0.058)	-0.019 (-0.36, 0.221)	0.16 (-0.075, 0.378)
0.24	-0.004 (-0.033, 0.028)	0.012 (-0.011, 0.038)	-0.032 (-0.388, 0.211)	0.091 (-0.099, 0.273)
0.25	0 (-0.034, 0.034)	0.014 (-0.006, 0.043)	0 (-0.394, 0.241)	0.111 (-0.048, 0.306)
0.26	0.005 (-0.028, 0.04)	0.014 (-0.006, 0.043)	0.036 (-0.297, 0.272)	0.11 (-0.054, 0.304)
0.27	0.01 (-0.024, 0.041)	0.014 (-0.006, 0.043)	0.077 (-0.269, 0.323)	0.109 (-0.061, 0.303)
0.28	0.009 (-0.026, 0.04)	0.014 (-0.007, 0.043)	0.07 (-0.283, 0.317)	0.107 (-0.067, 0.301)
0.29	0.012 (-0.021, 0.041)	0.014 (-0.007, 0.043)	0.091 (-0.252, 0.349)	0.106 (-0.074, 0.299)
0.30	0.018 (-0.014, 0.048)	0 (0, 0)	0.143 (-0.111, 0.417)	0 (0, 0)

[a]Different risk thresholds may be selected depending on the proposed intervention (i.e., balancing the risk/benefit of exposing false positives to an intervention to benefit the most true positives), as well as patient or clinician preference.
[b]Standardized net benefit is calculated as the net benefit / outcome prevalence, showing the proportion of improvement in net benefit at the selected risk threshold.

Part C: Supplementary Results Figures

Fig S5: Pooled Predicted Probabilities for PsyMetRiC in the Australian sample

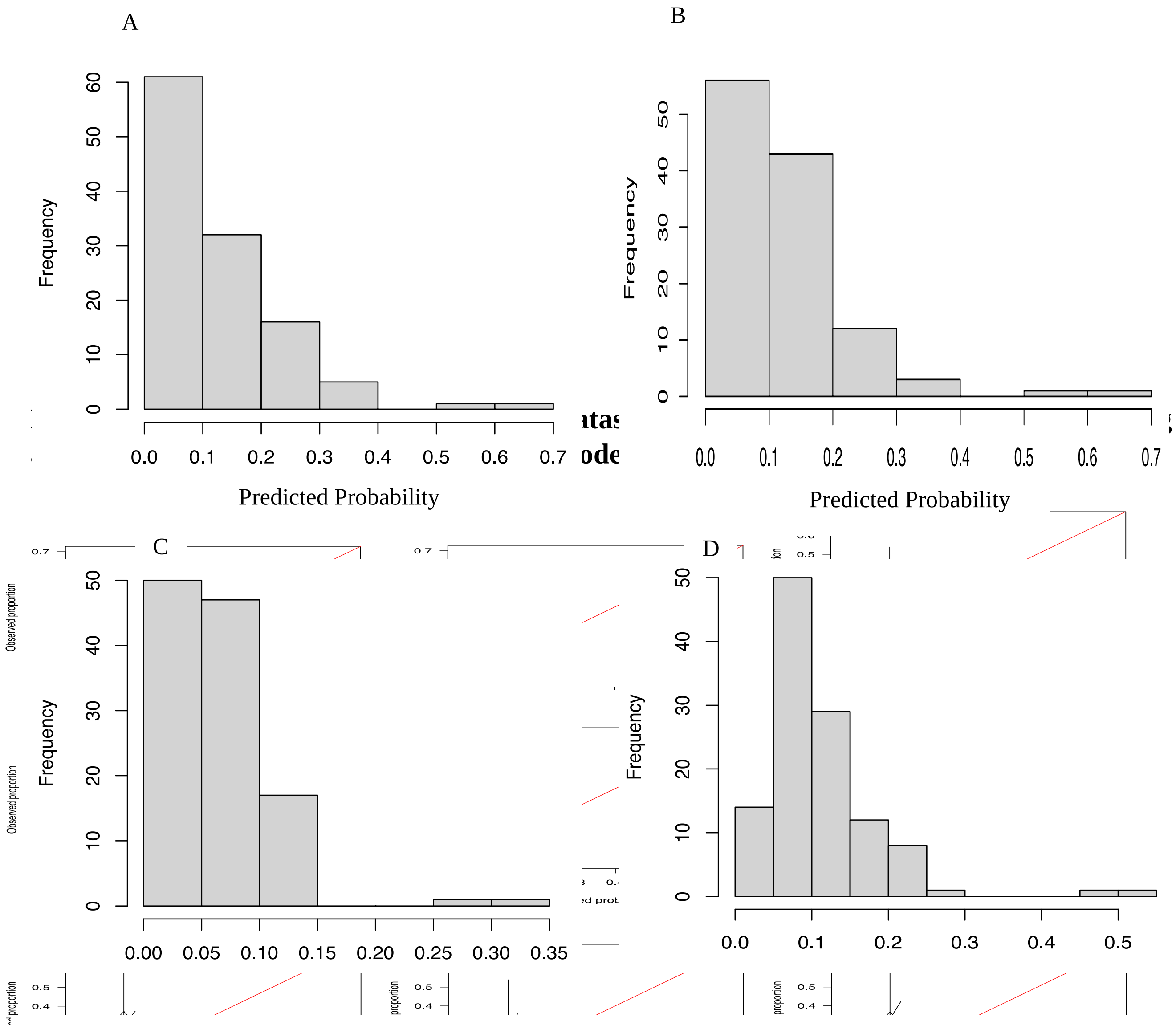

A = Full-Model (before updating and recalibration); B = Partial-Model (before updating and recalibration); C = Full-Model (after updating and recalibration); D = Partial-Model (after updating and recalibration)

Calibration plots illustrate agreement between observed risk (y axis) and predicted risk (x axis). Perfect agreement would trace the red line. Algorithm calibration is illustrated by the black line. Triangles denote grouped observations for participants at deciles of predicted risk, with 95% C.I.'s indicated by the vertical black lines.

Fig S6b: Calibration Plots Across Imputed Datasets in the Australian sample Before Updating and Recalibration for the PsyMetRiC Partial-Model.

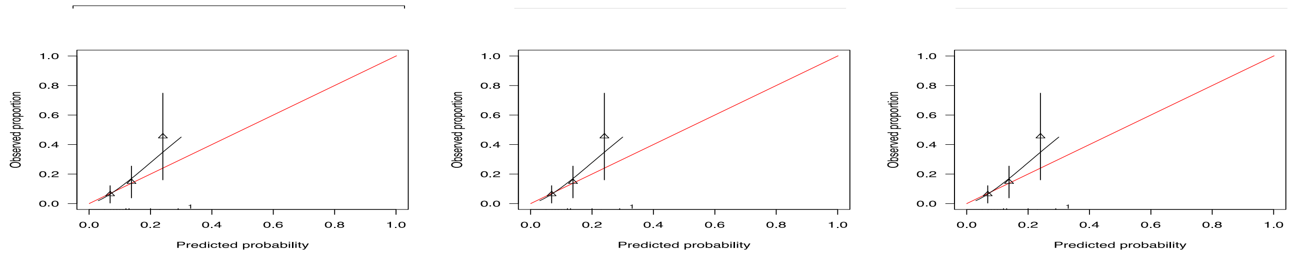

Calibration plots illustrate agreement between observed risk (y axis) and predicted risk (x axis). Perfect agreement would trace the red line. Algorithm calibration is illustrated by the black line. Triangles denote grouped observations for participants at deciles of predicted risk, with 95% C.I.'s indicated by the vertical black lines.

Fig S7a: Calibration Plots Across Imputed Datasets in the Australian sample After Updating

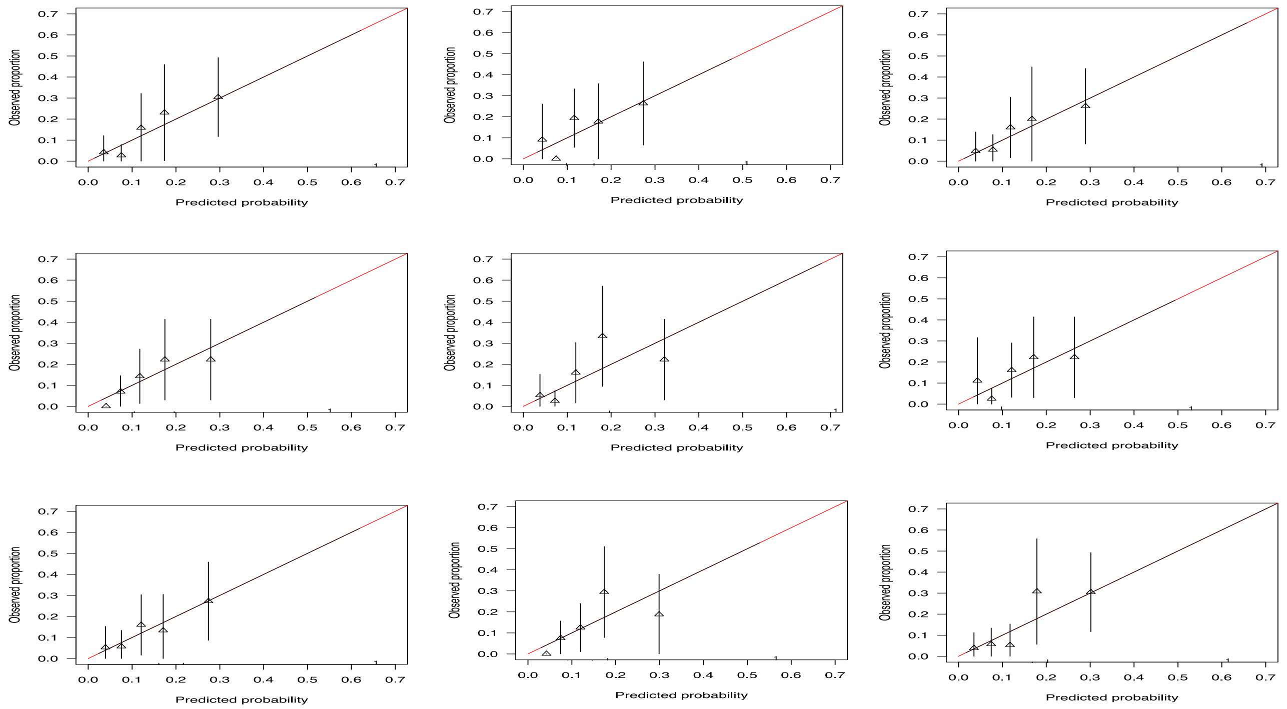

and Recalibration for the PsyMetRiC Full-Model.

Calibration plots illustrate agreement between observed risk (y axis) and predicted risk (x axis). Perfect agreement would trace the red line. Algorithm calibration is illustrated by the black line. Triangles denote grouped observations for participants at deciles of predicted risk, with 95% C.I.'s indicated by the vertical black lines.

Fig S7b: Calibration Plots Across Imputed Datasets in the Australian sample After Updating and Recalibration for the PsyMetRiC Partial-Model.

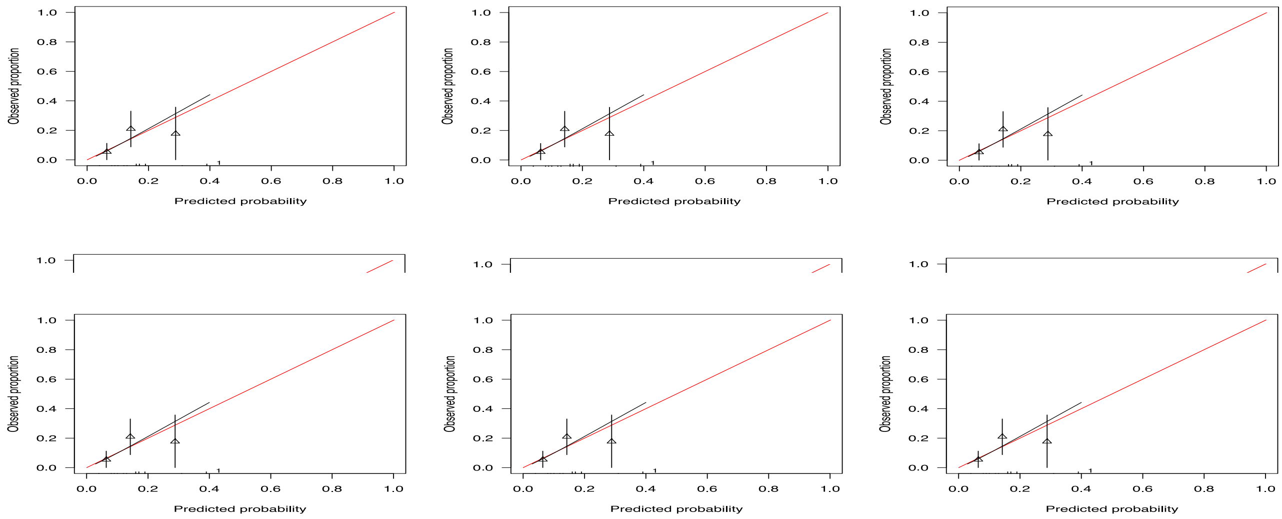

Calibration plots illustrate agreement between observed risk (y axis) and predicted risk (x axis). Perfect agreement would trace the red line. Algorithm calibration is illustrated by the black line. Triangles denote grouped observations for participants at deciles of predicted risk, with 95% C.I.'s indicated by the vertical black lines.

Fig S8a: Clinical Usefulness Across Imputed Datasets in the Australian Sample Before and After Updating and Recalibration for the PsyMetRiC Full-Model.

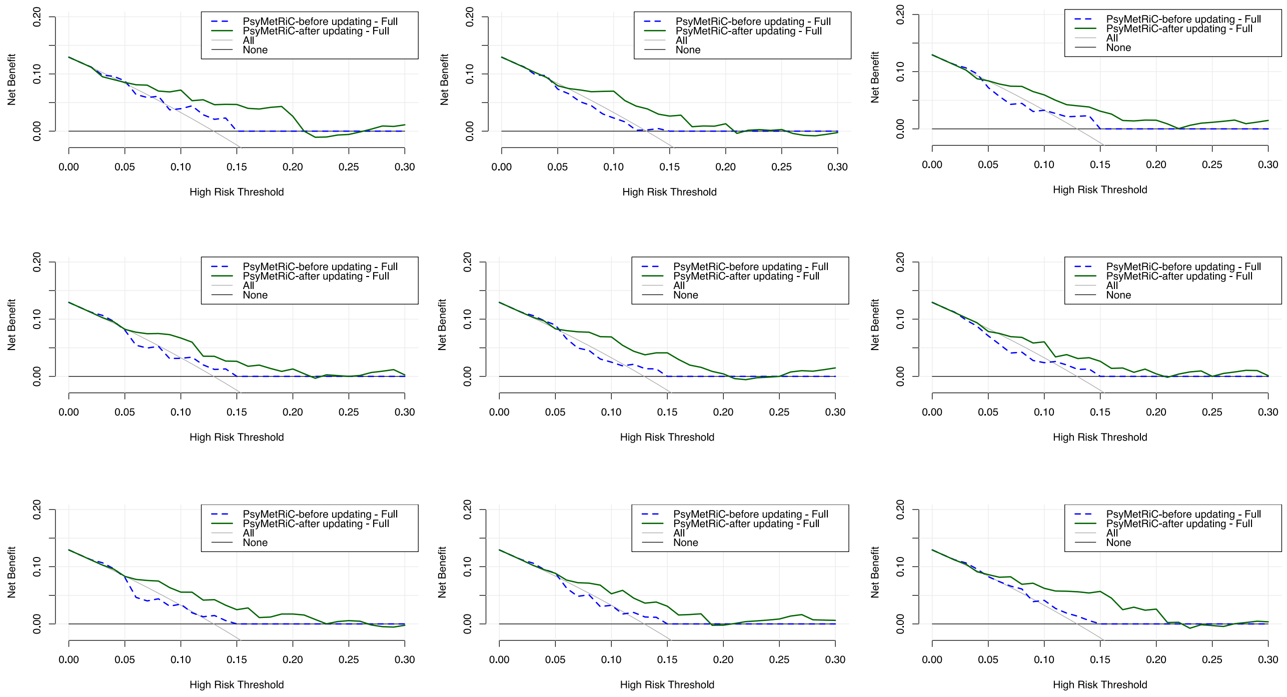

The plots report net benefit (y axis) of PsyMetRiC Full-Model (original = blue dotted line; updated and recalibrated = solid green line) across a range of reasonable risk thresholds (x axis) compared with intervening in all (grey line) or intervening in none (black line).

Fig S8b: Clinical Usefulness Across Imputed Datasets in the Australian Sample Before and

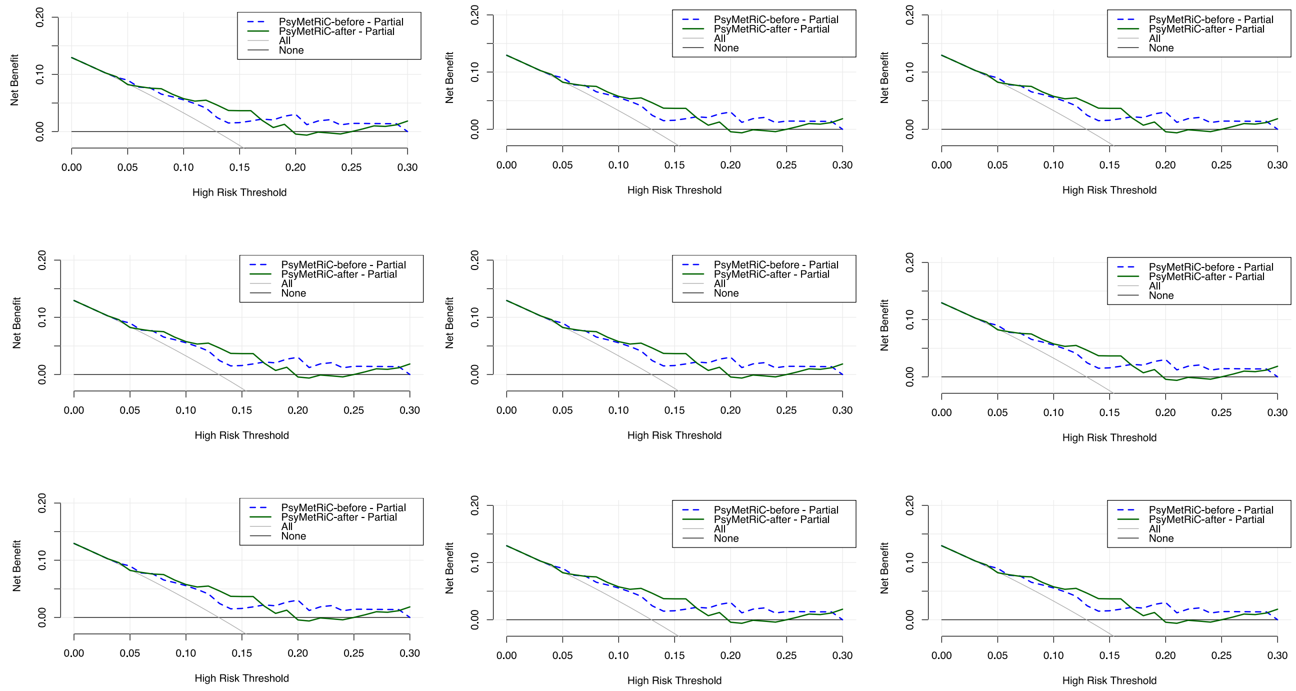

After Updating and Recalibration for the PsyMetRiC Partial-Model.

The plots report net benefit (y axis) of PsyMetRiC Partial-Model (original = blue dotted line; updated and recalibrated = solid green line) across a range of reasonable risk thresholds (x axis) compared with intervening in all (grey line) or intervening in none (black line).

Supplementary Data: Completed TRIPOD Checklist for Model Validation

Section/Topic	m	Checklist Item	Section
**Title and abstract**			
Title	1	Identify the study as developing and/or validating a multivariable prediction model, the target population, and the outcome to be predicted.	Title
Abstract	2	Provide a summary of objectives, study design, setting, participants, sample size, predictors, outcome, statistical analysis, results, and conclusions.	Abstract
**Introduction**			
Background and objectives	3a	Explain the medical context (including whether diagnostic or prognostic) and rationale for developing or validating the multivariable prediction model, including references to existing models.	Introduction
	3b	Specify the objectives, including whether the study describes the development or validation of the model or both.	Introduction
**Methods**			
Source of data	4a	Describe the study design or source of data (e.g., randomized trial, cohort, or registry data), separately for the development and validation data sets, if applicable.	Methods – Study Design and Setting
	4b	Specify the key study dates, including start of accrual; end of accrual; and, if applicable, end of follow-up.	Methods – Study Design and Setting
Participants	5a	Specify key elements of the study setting (e.g., primary care, secondary care, general population) including number and location of centres.	Methods – Study Design and Setting
	5b	Describe eligibility criteria for participants.	Methods - Participants
	5c	Give details of treatments received, if relevant.	Table 1
Outcome	6a	Clearly define the outcome that is predicted by the prediction model, including how and when assessed.	Methods - Outcome
	6b	Report any actions to blind assessment of the outcome to be predicted.	NA
Predictors	7a	Clearly define all predictors used in developing or validating the multivariable prediction model, including how and when they were measured.	Methods – Exposures; Supplementary File
	7b	Report any actions to blind assessment of predictors for the outcome and other predictors.	NA
Sample size	8	Explain how the study size was arrived at.	Methods –

			Statistical Analysis
Missing data	9	Describe how missing data were handled (e.g., complete-case analysis, single imputation, multiple imputation) with details of any imputation method.	Methods – Statistical Analysis; Supplementary File
Statistical analysis methods	10c	For validation, describe how the predictions were calculated.	Methods – Statistical Analysis; Supplementary File
	10d	Specify all measures used to assess model performance and, if relevant, to compare multiple models.	Methods – Statistical Analysis; Supplementary File
	10e	Describe any model updating (e.g., recalibration) arising from the validation, if done.	Methods – Statistical Analysis; Supplementary File
Risk groups	11	Provide details on how risk groups were created, if done.	NA
Developmen t vs. validation	12	For validation, identify any differences from the development data in setting, eligibility criteria, outcome, and predictors.	Table 1
**Results**			
Participants	13a	Describe the flow of participants through the study, including the number of participants with and without the outcome and, if applicable, a summary of the follow-up time. A diagram may be helpful.	Table 1
	13b	Describe the characteristics of the participants (basic demographics, clinical features, available predictors), including the number of participants with missing data for predictors and outcome.	Table 1
	13c	For validation, show a comparison with the development data of the distribution of important variables (demographics, predictors and outcome).	Table 1
Model performance	16	Report performance measures (with CIs) for the prediction model.	Table 2, Figures 1-2, Supplementary File
Model-updating	17	If done, report the results from any model updating (i.e., model specification, model performance).	Table 2, Figures 1-2, Supplementar y File
**Discussion**			
Limitations	18	Discuss any limitations of the study (such as nonrepresentative sample, few events per predictor, missing data).	Discussion - Limitations
Interpretatio n	19a	For validation, discuss the results with reference to performance in the development data, and any other validation data.	Discussion

	19b	Give an overall interpretation of the results, considering objectives, limitations, results from similar studies, and other relevant evidence.	Discussion
Implications	20	Discuss the potential clinical use of the model and implications for future research.	Discussion
**Other information**			
Supplementary information	21	Provide information about the availability of supplementary resources, such as study protocol, Web calculator, and data sets.	Methods – Statistical Analysis; Supplementary File
Funding	22	Give the source of funding and the role of the funders for the present study.	Funding

References

1. Alberti KGMM, Zimmet P, Shaw J. Metabolic syndrome—a new world- wide definition. A consensus statement from the international diabetes federation. *Diabetic medicine* 2006; **23**(5): 469-80.
2. Perry BI, Osimo EF, Upthegrove R, et al. Development and external validation of the Psychosis Metabolic Risk Calculator (PsyMetRiC): a cardiometabolic risk prediction algorithm for young people with psychosis. *Lancet Psychiatry* 2021; **8**(7): 589-98.
3. Leucht S, Cipriani A, Spineli L, et al. Comparative efficacy and tolerability of 15 antipsychotic drugs in schizophrenia: a multiple-treatments meta-analysis. *Lancet* 2013; **382**(9896): 951-62.
4. Bak M, Fransen A, Janssen J, van Os J, Drukker M. Almost all antipsychotics result in weight gain: a meta-analysis. *PLoS One* 2014; **9**(4): e94112.
5. Matar HE, Almerie MQ, Makhoul S, Xia J, Humphreys P. Pericyazine for schizophrenia. *Cochrane Database Syst Rev* 2014; (5): CD007479.
6. Pillinger T, McCutcheon RA, Vano L, et al. Comparative effects of 18 antipsychotics on metabolic function in patients with schizophrenia, predictors of metabolic dysregulation, and association with psychopathology: a systematic review and network meta-analysis. *Lancet Psychiatry* 2020; **7**(1): 64-77.
7. Alonso-Pedrero L, Bes-Rastrollo M, Marti A. Effects of antidepressant and antipsychotic use on weight gain: A systematic review. *Obes Rev* 2019; **20**(12): 1680-90.
8. White IR, Royston P, Wood AM. Multiple imputation using chained equations: Issues and guidance for practice. *Stat Med* 2011; **30**(4): 377-99.
9. Lee HJ, Huber, J.,. Multiple imputation with large proportions of missing data: How much is too much? United Kingdom Stata Users' Group Meetings 2011; 2011: Stata Users Group; 2011.
10. Dong Y, Peng CY. Principled missing data methods for researchers. *Springerplus* 2013; **2**(1): 222.
11. Fukuma S, Shimizu S, Shintani A, Kamitani T, Akizawa T, Fukuhara S. Development and validation of a prediction model for loss of physical function in elderly hemodialysis patients. *Nephrol Dial Transplant* 2018; **33**(8): 1452-8.
12. Copas JB. Regression, prediction and shrinkage. *Journal of the Royal Statistical Society: Series B* 1983; **45**: 311-54.
13. Vickers AJ, Elkin EB. Decision curve analysis: a novel method for evaluating prediction models. *Med Decis Making* 2006; **26**(6): 565-74.
14. Vickers AJ, van Calster B, Steyerberg EW. A simple, step-by-step guide to interpreting decision curve analysis. *Diagn Progn Res* 2019; **3**: 18.
15. Pavlou M, Qu C, Omar RZ, et al. Estimation of required sample size for external validation of risk models for binary outcomes. *Stat Methods Med Res* 2021; **30**(10): 2187-206.

16. Riley RD, Snell KI, Ensor J, et al. Minimum sample size for developing a multivariable prediction model: PART II - binary and time-to-event outcomes. *Stat Med* 2019; **38**(7): 1276-96.
